# Anesthetics Impact the Resolution of Inflammation

**DOI:** 10.1371/journal.pone.0001879

**Published:** 2008-04-02

**Authors:** Nan Chiang, Jan M. Schwab, Gabrielle Fredman, Kie Kasuga, Simon Gelman, Charles N. Serhan

**Affiliations:** Center for Experimental Therapeutics and Reperfusion Injury, Department of Anesthesiology, Perioperative and Pain Medicine, Brigham and Women's Hospital and Harvard Medical School, Boston, Massachusetts, United States of America; Emory University, United States of America

## Abstract

**Background:**

Local and volatile anesthetics are widely used for surgery. It is not known whether anesthetics impinge on the orchestrated events in spontaneous resolution of acute inflammation. Here we investigated whether a commonly used local anesthetic (lidocaine) and a widely used inhaled anesthetic (isoflurane) impact the active process of resolution of inflammation.

**Methods and Findings:**

Using murine peritonitis induced by zymosan and a systems approach, we report that lidocaine delayed and blocked key events in resolution of inflammation. Lidocaine inhibited both PMN apoptosis and macrophage uptake of apoptotic PMN, events that contributed to impaired PMN removal from exudates and thereby delayed the onset of resolution of acute inflammation and return to homeostasis. Lidocaine did not alter the levels of specific lipid mediators, including pro-inflammatory leukotriene B_4_, prostaglandin E_2_ and anti-inflammatory lipoxin A_4_, in the cell-free peritoneal lavages. Addition of a lipoxin A_4_ stable analog, partially rescued lidocaine-delayed resolution of inflammation. To identify protein components underlying lidocaine's actions in resolution, systematic proteomics was carried out using nanospray-liquid chromatography-tandem mass spectrometry. Lidocaine selectively up-regulated pro-inflammatory proteins including S100A8/9 and CRAMP/LL-37, and down-regulated anti-inflammatory and some pro-resolution peptides and proteins including IL-4, IL-13, TGF-â and Galectin-1. In contrast, the volatile anesthetic isoflurane promoted resolution in this system, diminishing the amplitude of PMN infiltration and shortening the resolution interval (R*i*) ∼50%. In addition, isoflurane down-regulated a panel of pro-inflammatory chemokines and cytokines, as well as proteins known to be active in cell migration and chemotaxis (i.e., CRAMP and cofilin-1). The distinct impact of lidocaine and isoflurane on selective molecules may underlie their opposite actions in resolution of inflammation, namely lidocaine delayed the onset of resoluion (T_max_), while isoflurane shortened resolution interval (R*i*).

**Conclusions:**

Taken together, both local and volatile anesthetics impact endogenous resolution program(s), altering specific resolution indices and selective cellular/molecular components in inflammation-resolution. Isoflurane enhances whereas lidocaine impairs timely resolution of acute inflammation.

## Introduction

Resolution of acute inflammation was widely held to be a passive event [1]. It is now clear that tissue resolution or its return from an inflammatory and/or disease state is an active process involving novel mediators [2], [3]. Non-resolved inflammation can exacerbate tissue injury and may cause functional damage via abscess or scar formation [1]. An emerging body of evidence now indicates that anti-inflammation (i.e. inhibiting the cardinal signs of inflammation [1]) and pro-resolution, namely activating endogenous resolution programs [3] are distinct mechanisms in the control of inflammation [3,and for a recent consensus report, see ref 4]. Classic antiinflammatories are enzyme inhibitors and/or receptor antagonists such as inhibitors of cyclooxygenases (COX) and antagonists for leukotriene (LT) receptors. Resolution agonists, in comparison, are also antiinflammatories, but act by different mechanisms than the classic ones [recently reviewed in ref 5].

Resolution agonists (such as lipoxins), for example, are agonists that not only block neutrophil (PMN) actions [5], but also stimulate non-phlogistic monocyte recruitment [6] and macrophage uptake of apoptotic PMN [7]. Hence resolution agonists have two main mechanisms of actions at the tissue level; they lower the numbers of infiltrating PMN to the inflamed sites and tissues; and they stimulate the active removal of debris and apoptotic PMN from the inflamed sites by non-phlogistic activation of macrophages [5]. Because it is important to study resolution of inflammation as a distinct process, we introduced resolution indices to a) quantitate the overall process; b) access the roles of specific mediators; and c) pinpoint mechanisms of pharmacological interventions in the resolution of inflammation.

To characterize resolution of inflammation in cellular and molecular terms, we established a resolution map and defined the main quantitative indices (Fig. S1A) [8]. These indices chart and take into account (i) the *magnitude* of PMN tissue infiltration (maximal PMN, Ψ_max_); (ii) the time interval when numbers of PMN reach Ψ_max_ within exudates (T_max_); (iii) *duration*: the time point (T_50_) when PMN numbers reduce to 50% of Ψ_max_ (R_50_); and (iv) the *resolution interval* (R*_i_*): the time interval from the maximum PMN point (Ψ_max_) to the 50% reduction point (R_50_) [*i.e.* T_50-_T_max_]. Using this set of resolution indices, we demonstrated that endogenous mediators such as resolvins and protectins accelerate resolution as evidenced by initiating the resolution of inflammation at earlier times (↓T_max_ and T_50_) and/or shortening the resolution interval (↓R*_i_*) [8], [9]. The actions of these pro-resolution mediators sharply contrast those of agents and currently used therapeutics that are inhibitors and “resolution toxic”. These drugs/agents have an unwanted impact on resolution such as inhibitors of COX-2 [9], [10] and lipoxygenases (LOX) [9]. Thus, this set of resolution indices can be utilized to evaluate the impact of endogenous mediators as well as potential new therapeutic agents in inflammatory resolution because they reflect the summation of tissue-level events that are multi-level cellular and molecular processes in resolution of inflammation.

Surgery itself initiates an inflammatory response [11], and local anesthetics, both topical and volatile, are widely used during surgery [12]. Some anesthetics (i.e., lidocaine and isoflurane) are reported to reduce inflammatory markers, including cytokines and chemokines [13], [14]. Potential impact of these widely used anesthetics on resolution of inflammation has not been established. The actions of many widely used current drugs in the pharmacopeia on resolution of inflammation remain unknown because their resolution characteristics were not evaluated at the time of their classical development. Appropriate qualified models of resolution were simply not yet available [8], [9].

Here, we report using an unbiased systems approach that the widely used local anesthetic, lidocaine, and a widely used volatile anesthetic, isoflurane, each impact in vivo the resolution of acute inflammation in opposite directions that were quantified using resolution indices. We also characterize their multi-level impact on key cellular and molecular components in resolution of inflammation.

## Results

### Local anesthetic lidocaine impairs resolution

**Figure 1:**
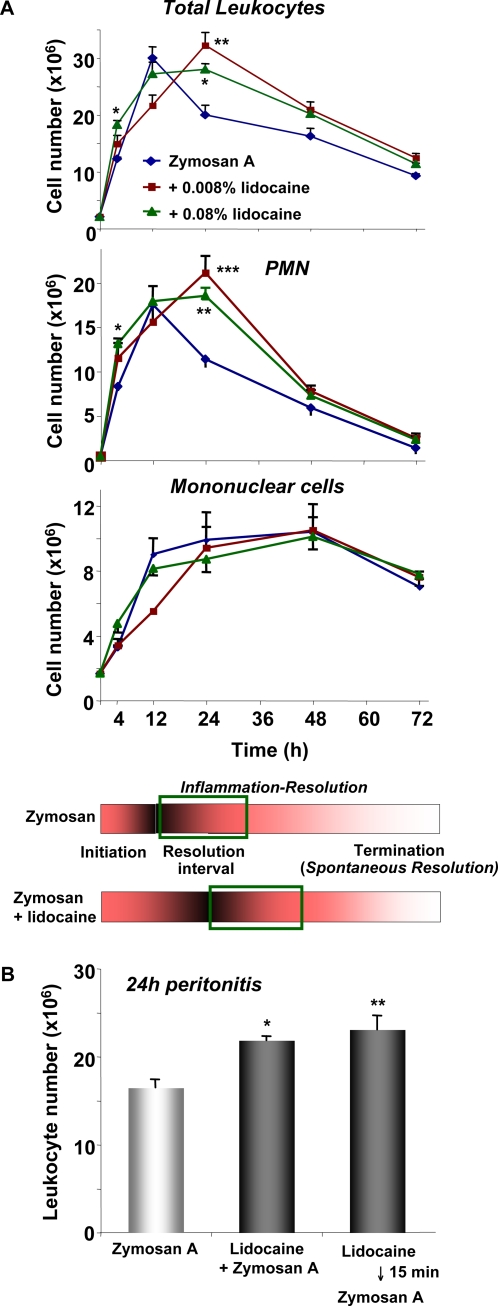
Lidocaine alters leukocyte infiltration during acute inflammation and delays resolution. (A) Mice were injected with zymosan A in the absence or presence of lidocaine (0.008% or 0.08%) and peritoneal lavages were collected at indicated time points. Total leukocytes were enumerated by light microscopy, and PMN and mononuclear cells determined by differential leukocyte counting. Results are expressed as the mean±SEM from n = 3–4. *p<0.05, **p<0.01, ***p<0.001 when compared to mice treated with zymosan A alone at the same time points. (B) Mice were injected with lidocaine (0.08%) 15 min prior to injection of zymosan A. Peritoneal lavages were collected at 24 h, and total leukocytes enumerated. Results are expressed as mean±SEM from n = 3. *p<0.05, **p<0.01 when compared to mice treated with zymosan A alone.

We first determined whether lidocaine alters cellular infiltration in a self-limited spontaneously resolving murine peritonitis. For these analyses, we used our reported resolution map that was constructed using an unbiased systems approach that combined cell trafficking into inflammatory exudates and mass spectrometry-based proteomics and lipid mediator lipidomics of resolving exudates [8]. Here, a microbial stimulus, the yeast wall zymosan A, was administered intraperitoneally to initiate inflammation [15], together with lidocaine given concomitantly. Given the inflammation-resolution map as a background terrain, lidocaine was introduced in order to determine if it significantly changed the signature of resolution map and indices in zymosan-initiated peritonitis. Inflammatory exudates were collected at the indicated time intervals 4–72 h (Fig. 1A). Zymosan alone, as expected, stimulated an acute increase in the total leukocyte numbers (i.e. PMN and mononuclear cells) present in the peritoneal exudates during the initial phase of inflammation (4 h after zymosan, 11.8±0.4×10^6^ leukocytes), with a maximal infiltration at 12 h (30.0±2.5×10^6^ leukocytes), followed by a decline or resolution as monitored to 72 h. The time course of PMN infiltration followed a similar trend, peaking (17.5±2.5×10^6^ PMN) at 12 h after zymosan challenge (Fig. 1A). An anesthetic dose of lidocaine, i.e. 0.08% (w/v) [16] administered with zymosan A significantly increased the number of total leukocytes by ∼49% within exudates at 4 h (*p*<0.05). The increase in exudate PMN was ∼58% (*p*<0.05). In the mice treated with both lidocaine and zymosan, the numbers of PMN continued to increase after 12 h and reached a maximum at 24 h. As a result, in the presence of lidocaine, the number of PMN in the exudate was significantly increased at this time point (∼60% increase, *p*<0.01). In contrast, the patterns of mononuclear cell infiltrates did not appear to be significantly altered by lidocaine treatment in this time course (4–72 h). Even doses as low as 0.008% (w/v) lidocaine, when given together with zymosan, led to a significant increase in the accumulation of PMN at 24 h (∼75% increase, *p*<0.001). Lidocaine alone without zymosan challenge did not alter peritoneal leukocyte numbers in this 4–24 h interval after administration (Fig. S1B). These results suggested that lidocaine might hamper PMN clearance during the normal spontaneous resolution phase of acute inflammation.

As shown in Figures 1A and S1C, in the zymosan-initiated peritonitis, the number of PMN reached a maximum at 12 h. The time intervals between 12 h (T_max_) and 35 h (T_50_), exudate PMN decreased in number from 17.5×10^6^ PMN (Ψ_max_; maximal PMN number) to 8.8×10^6^ PMN (R_50_; essentially 50% reduction of PMN). This period of neutrophilic loss from the exudates is termed the *resolution interval* (R*_i_*) [8]. In mice treated with zymosan alone, R*_i_* was ∼23 h (*i.e*., 12–35 h). When the resolution indices were calculated with lidocaine treatment together with zymosan, it was apparent that both the anesthetic (0.08%) and sub-anesthetic doses (0.008%) of lidocaine increased Ψ_max_ and shifted the onset of R*_i_* from 12 h to a later time point (T_max = _24 h) (see Fig. S1C and *vide infra*). These results demonstrate that lidocaine directly delayed the spontaneous resolution of zymosan-initiated acute inflammation. Especially, lidocaine increased the dwell time of PMN present within the exudates, possibly blocking the clearance of PMN from the exudates in vivo (see below).

Surgery can induce local inflammation via tissue injury, and lidocaine is usually given before surgery [11], [12]. In order to mimic such a clinical scenario, mice were treated with lidocaine (0.08%) 15 min before initiation of acute inflammation by zymosan. This prior exposure to lidocaine significantly potentiated zymosan-initiated leukocyte infiltration at 24 h by ∼40% (*cf*. zymosan alone, *p*<0.01). This is similar to the results obtained with mice that received lidocaine and zymosan together, which gave a ∼33% increase in the number of leukocytes present in the exudates when compared to zymosan alone (*p*<0.05, Fig. 1B). Thus, lidocaine administration, either just before or concomitant with zymosan, caused significant increases in the number of PMN present in exudates in the resolution phase of acute inflammation.

**Figure 2:**
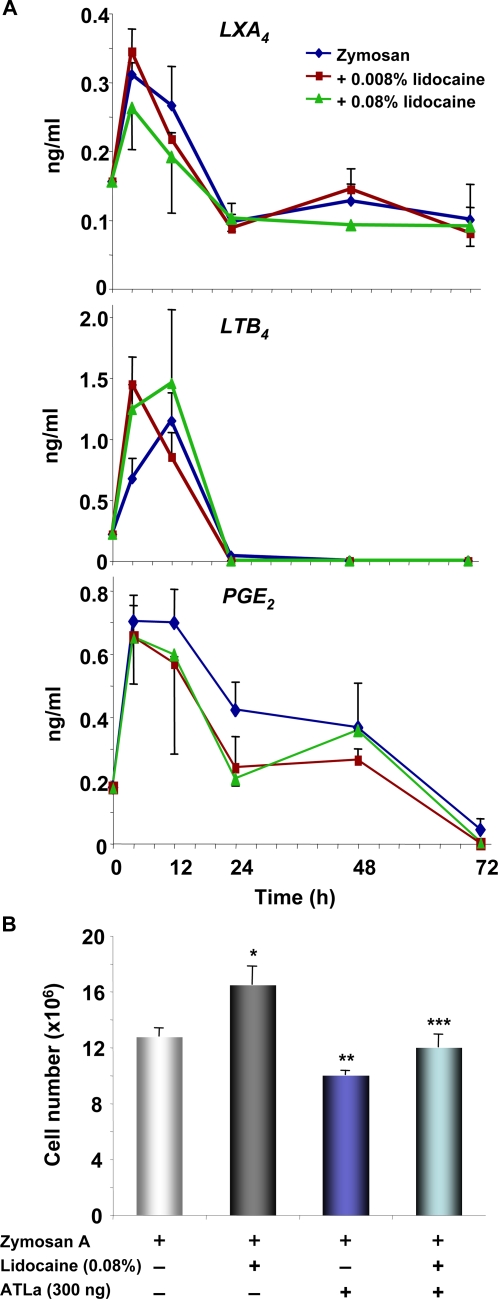
Lidocaine did not directly alter selective eicosanoid levels in cell-free exudates: LXA_4_ rescues lidocaine-delayed resolution. (A) Cell-free lavages from murine peritoneum were collected at indicated time points after zymosan challenge (1 mg/ml). LXA_4_, LTB_4_ and PGE_2_ amounts were determined by ELISA. Results are expressed as the mean±SEM from duplicates of n = 3, and were expressed as amounts (ng/ml). (B) Mice were injected with zymosan A together with lidocaine (0.08%), ATLa (300 ng), or lidocaine plus ATLa. Peritoneal lavages were collected at 24 h, and total leukocytes enumerated. Results are expressed as mean±SEM from n = 3. *p = 0.03 **p = 0.01 when compared to mice treated with zymosan A alone. ***p = 0.04 when compared to mice treated with zymosan A and lidocaine.

Specialized lipid mediators play a key role in resolution of inflammation [5] with some specifically switched on during the resolution phase to promote resolution [17]. Here, key lipid mediators were monitored in murine exudates, including lipoxin (LX) A_4_, an anti-inflammatory and pro-resolution mediator, and the pro-inflammatory LTB_4_ and prostaglandin (PG) E_2_. In this system, the maximal levels present in cell-free lavages of the exudates of both LTB_4_ and LXA_4_ were obtained at 4 h. These subsequently subsided within 24 h (Fig. 2A). Lidocaine did not significantly alter the levels of LXA_4_, LTB_4_ or PGE_2_ present in these cell-free lavages of the peritoneal exudates. Thus, these eicosanoids likely reflect the profile from resident peritoneal cells including macrophages as are less likely to report eicosanoids generated by the infiltrating leukocytes.

Lipoxins are potent agonists for resolution of inflamed tissues by regulating leukocyte infiltration, stimulating macrophage clearance of apoptotic PMN and also their exit via lymphatics [5], [8], [9]. Since LXA_4_ can rescue inhibitor-imposed lesion with, for example, a selective COX-2 inhibitor [9], we questioned whether these resolution agonists impact leukocyte infiltration in lidocaine-treated mice. At 24 h, lidocaine (0.08%, ∼0.8 mg) administration increased, while ATLa (a stable analog for aspirin-triggered 15-epi-lipoxin A_4_, 300 ng, i.p.) decreased exudates cell numbers, when compared with mice treated with zymosan alone. When ATLa was administered along with lidocaine and zymosan, it significantly reduced exudate leukocytes compared to mice received lidocaine and zymosan (*p* = 0.04) (Fig. 2B). Thus, pro-resolution mediators, such as lipoxins, at much lower doses (by more than 3 log orders) partially rescued the defective resolution of inflammation caused by lidocaine.

### Lidocaine impairs PMN apoptosis and their removal by macrophages

**Figure 3:**
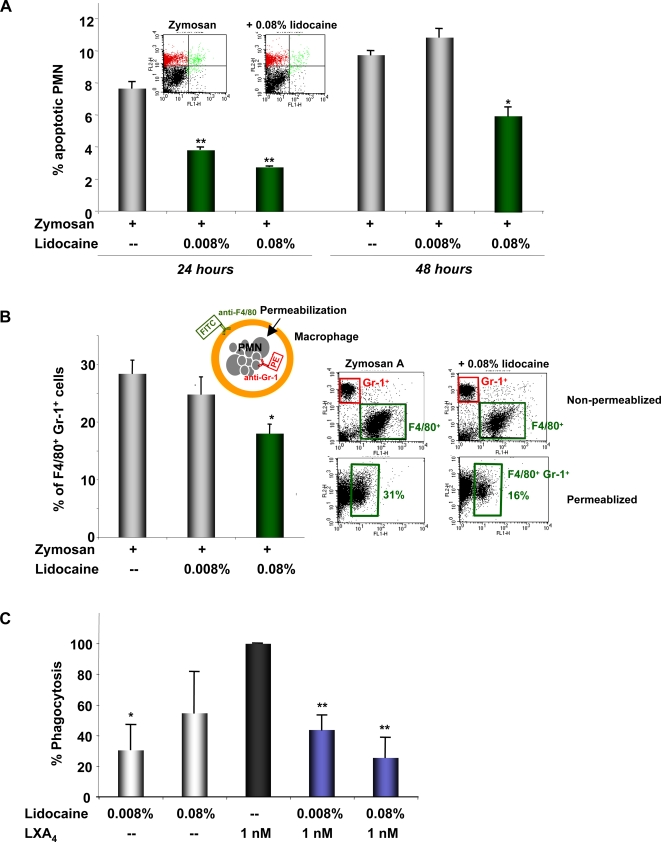
Lidocaine impairs PMN apoptosis and macrophage ingestion of PMN in vivo and zymosan in vitro. (A) *Apoptosis in vivo*. Peritoneal cells were collected at 24 h or 48 h and labeled with FITC-annexin-V and PE-conjugated anti-Gr-1 Ab. The apoptotic PMN (annexin-V^+^Gr-1^+^) are expressed as % of total PMN (Gr-1^+^). Results are the mean±SEM from n = 3–4. *p<0.01, **p<0.001. (B) *Phagocytosis in vivo*. (*right*) Representative dot plots of FACS analysis. In the non-permeabilized lavage cells, Gr-1^+^ represents PMN, and F4/80^+^ represents macrophages; and in the permeabilized cells, F4/80^+^Gr-1^+^ cell population represents macrophages with ingested PMN. (*left*) Results are expressed as the mean±SEM from n = 3–4, and were expressed as percent of the F4/80^+^Gr-1^+^ cells. *p<0.05. (C) *Phagocytosis in vitro*. Murine peritoneal resident macrophages were incubated with indicated compounds or vehicle alone for 20 min followed by addition of FITC-zymosan at a 10:1 ratio for 30 min. Cells were then quenched and fluorescence determined. Phagocytosis activity in the presence of 1 nM of LXA_4_ was taken as 100%. Results are expressed as the mean±SEM from n = 3–4, and were expressed as % phagocytosis. *p<0.05, **p<0.01, compared to LXA_4_ alone.

PMN apoptosis and their subsequent removal by macrophages are essential components of resolution at the tissue level [1], [18]. Since lidocaine delayed PMN clearance in the resolution phase, we considered that lidocaine might have an impact on PMN apoptosis. To address this, peritoneal cells were collected at 24 h after zymosan challenge, well within the resolution phase, and labeled with FITC-annexin-V and PE-conjugated anti-Gr-1 Ab, a specific cell surface marker for mouse PMN. Peritoneal cells collected from mice receiving lidocaine (at both 0.08% and 0.008%) together with zymosan showed significantly decreased annexin-V^+^Gr-1^+^ cells by 50% and 64%, respectively, indicating reduced PMN apoptosis (Fig. 3A). At 48 h after zymosan challenge, lidocaine at 0.08% also reduced PMN apoptosis ∼40% (*p*<0.01).

We next determined whether lidocaine impacts macrophage ingestion of PMNs. To this end, we carried out a phagocytosis-based analysis *in vivo* (Fig. 3B). Exudate cells were collected at 24 h after zymosan challenge, and macrophages were labeled with the FITC-conjugated anti-F4/80 Ab. This was followed by permeabilization of these cells, allowing labeling of ingested PMN with PE-conjugated anti-Gr-1 Ab. Cells with positive staining of both F4/80 and Gr-1 were then monitored by FACS analysis. Of interest, cells collected from mice treated with lidocaine (0.08%) together with zymosan showed significantly reduced F4/80^+^Gr-1^+^ cells (18.0±1.2%) when compared to those given only zymosan (28.4±1.6%) (*p*<0.05, Fig. 3B). The low dose of lidocaine (0.008%) also gave decreased F4/80^+^Gr-1^+^ cells (24.8±2.1%), albeit not significantly different from mice receiving zymosan alone. These results indicate that clinically used doses of lidocaine inhibit macrophage ingestion of apoptotic PMN *in vivo*, blocking their removal and resolution.

We also investigated whether lidocaine has a direct impact on isolated macrophages. To this end, we carried out *in vitro* phagocytosis of zymosan particles. This system represents recognition of microbes by the innate immune system [19]. Recently, we found that pro-resolution mediators such as LXA_4_ are potent stimulators of macrophage uptake of microbial particles, i.e., opsonized zymosan [9], in addition to stimulating the uptake of apoptotic PMN [7]. Of interest, lidocaine at both doses (0.008% and 0.08%), when added together with LXA_4_, significantly impaired LXA_4_-stimulated phagocytosis (Fig. 3C). Thus, lidocaine can be considered “resolution toxic” because it impairs key components at the level of tissue resolution, namely PMN apoptosis and macrophage phagocytosis, and blocks the protective action of LXA_4_.

### Lidocaine regulates both anti- and pro-inflammatory proteins: proteomics

**Figure 4:**
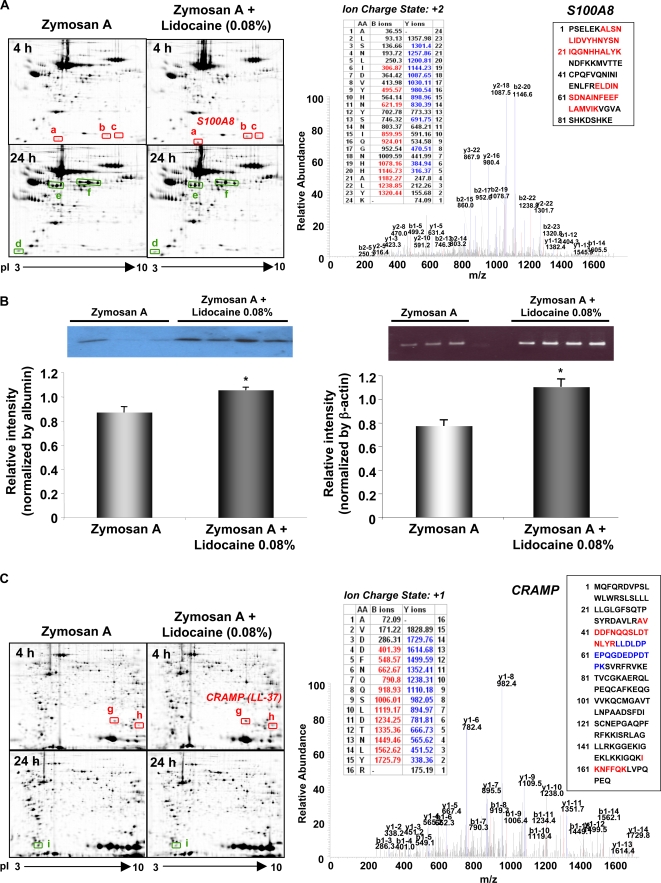
Lidocaine alters pro- and anti-inflammatory proteins: proteomics and cellular proteins. Mice were injected with zymosan A in the absence or presence of lidocaine. Both lavage fluids (A) and cell pellets (C) were collected at indicated time points and proteins separated by two-dimensional gel electrophoresis. Changes in individual protein levels were measured by image analysis. Selected proteins that display significant differences between treatments are indicated by arrows, and identified by LC/MS/MS and peptide mapping (see Materials and Methods). (B) (Left) S100 A9 protein levels. Supernatants from peritoneal lavages were subjected to Western blot analysis using an anti-S100A9 antibody. Relative intensities of immunoreactive bands were quantitated and normalized by albumin levels using an anti-albumin antibody. Data are expressed as mean±SEM from n = 3–4. *p = 0.02. (Right) S100 A9 mRNA levels. Peritoneal cells were collected and total RNA isolated for RT-PCR analysis using specific primers for mouse S100A9. Relative intensities of RT-PCR products were quantitated and normalized by β-actin message levels. Data are expressed as mean±SEM from n = 3–4. *p<0.01.

Using mass spectrometry-based resolution proteomics, we recently identified several components in inflammatory exudates, including haptoglobin, S100A9 and α1-macroglobulin, that may play active roles in promoting resolution of inflammation [8]. These proteins were identified by peptide mapping of in-gel digested proteins using capillary liquid chromatography-nanospray ion trap tandem mass spectrometry (nanospray-LC-MS-MS) and bioinformatics software (see Methods). Among these, S100A9 was present in exudates within 4 h of initiating inflammation and reached maximum levels at the onset of R*_i_* (12 h). These changes in S100A9 paralleled the time course of PMN infiltration (Fig. 1A) [8]. Both S100A8 and S100A9 are known to be abundant cytosolic proteins in human PMN that can be secreted and exhibit potent actions in inflammatory cell recruitment [20]. Also, S100 proteins belong to a new group of damage-associated molecular pattern proteins and may function as "alarm/danger" signals to propagate inflammation [21]. To determine whether lidocaine impacts these proteins during inflammation-resolution, we carried out temporal-differential analysis of peritoneal exudate proteins collected from zymosan-challenged mice in the presence or absence of the anesthetic dose of lidocaine (0.08%). Two time intervals were selected for analysis: one at 4 h within the early inflammatory phase, and the second at 24 h within the resolution phase, since lidocaine gave the most dramatic impact on PMN infiltration at these two time points (see Fig. 1A). Four hours after zymosan challenge, both S100A8 and S100A9 were significantly increased in the presence of lidocaine (Fig. 4A and Table 1). This increase was verified from mice that received lidocaine together with zymosan by Western blot analysis that also demonstrated an increase in S100A9 proteins in exudates (Fig. 4B). Also S100A9 mRNA levels were higher in these mice compared to mice that received zymosan alone as determined with RT-PCR (Fig. 4B). Thus, it is likely that S100A8/A9 complexes reflect, at least in part, the increases in PMN obtained in mice challenged with lidocaine and zymosan at 4 hours, compared to mice received zymosan alone.

**Table 1:** Exudate and cellular proteins regulated by lidocaine during inflammation-resolution

Spot	Protein	Time (h)	# identified peptides	Zymosan A	Zymosan A+Lidocaine	P value
* Exudate protein*						
a	S100A8	4	2	0.18±0.07	0.39±0.08 (↑117%)	0.02
b	S100A8	4	2	0.11±0.02	0.42±0.10 (↑282%)	0.01
c	S100A9	4	2	0.07±0.05	0.23±0.06 (↑229%)	0.02
d	Apolipoprotein CIII	24	2	0.32±0.03	0.22±0.02 (↓31%)	0.01
e	Fibrinogen γ polypeptide	24	8	2.32±0.04	1.87±0.17 (↓20%)	0.01
f	Fibrinogen β polypeptide	24	16	3.97±0.36	2.59±0.36(↓35%)	0.01
* Cellular protein*						
g	CRAMP (LL-37)	4	2	0.27±0.08	0.41±0.04 (↑52%)	0.05
h	CRAMP (LL-37)	4	3	0.14±0.06	0.29±0.05 (↑107%)	0.03
i	Galectin 1	24	2	0.49±0.06	0.23±0.08 (↓53%)	0.01

The proteins were identified by mass spectrometry (see Materials and Methods).

We also determined, in parallel with exudate cells, changes in cell-associated proteins from these mice. As shown in Figure 4C, lidocaine together with zymosan at 4 h gave significant up-regulation of several selective proteins compared to zymosan-challenged mice. Among them, CRAMP (cathelin-related anti-microbial peptide), the mouse homolog of anti-microbial protein LL-37, was increased approximately two-fold (Table 1). CRAMP is also a documented chemotactic factor for PMN, monocytes, mast cells, and T cells [22]. Thus, exudate CRAMP/LL-37 may also contribute to increased PMN numbers obtained at 4 h in lidocaine-treated mice (Fig. 1A). Moreover, we found that, within resolution at 24 h, lidocaine down-regulated galectin-1 ∼50% (Fig. 4C and Table 1). Galectin-1 inhibits PMN migration during PMN-endothelial interactions in vitro and in vivo [23]. In addition, Galectin-1 prolongs exposure of phosphatidylserine on the surface of leukocytes, suggesting a role in promoting PMN clearance [24]. Therefore, decreases in Galectin-1 levels from lidocaine-treated mice during resolution (i.e. 24 h) might also contribute to delayed PMN clearance, resulting in increased PMN dwell times in exudates (Fig. 1A).

### Lidocaine impacts chemical mediators in exudates

**Figure 5:**
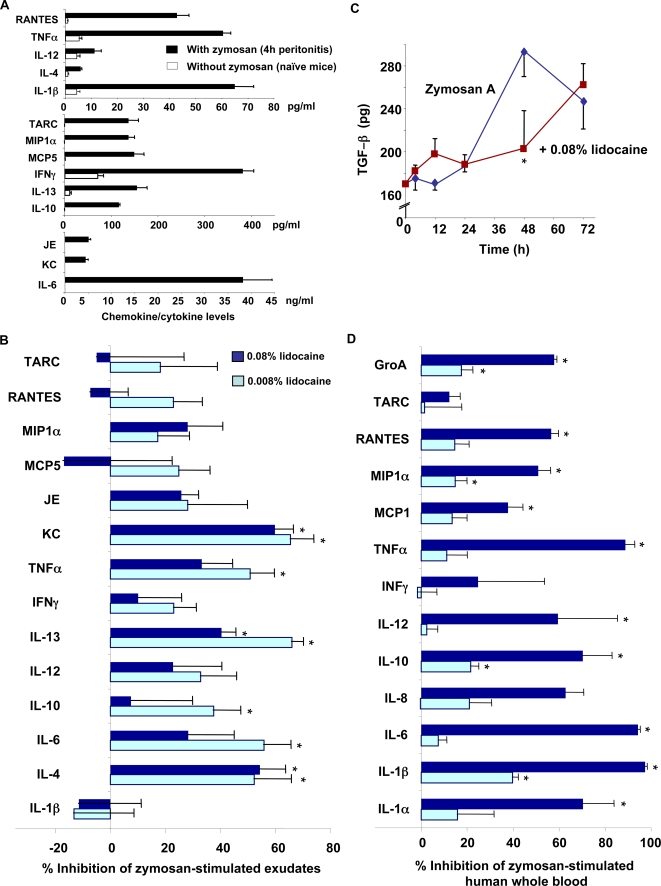
Lidocaine regulates selective pro- and anti-inflammatory cytokines/chemokines. (A, B) Mice were injected with zymosan alone or together with lidocaine (0.008% or 0.08%) for 4 h, and peritoneal cell-free lavage fluids collected. Cytokines and chemokines were expressed as (A) pg/ml or ng/ml in naïve mice and mice treated with zymosan alone, (B) percent inhibition of zymosan A-induced cytokine/chemokine levels by lidocaine. The amounts of cytokines and chemokines levels were determined by multiplexed sandwich ELISA. (C) TGF-β (active form) levels were determined by ELISA. (D) Human heparinized whole blood was incubated with either 0.008% or 0.08% of lidocaine in the presence of zymosan A (100 µg/ml) for 4 h, and plasma was collected. The amounts of cytokines and chemokines levels were determined by multiplexed sandwich ELISA. Results are the mean from duplicate determinations of n = 3–4. *p<0.05 when compared to mice treated with zymosan A alone (B, C) or human whole blood incubated with zymosan A alone (D).

Production of both pro-inflammatory (e.g. IL-1β, IL-6, IL-12, TNF-α) and anti-inflammatory (e.g. IL-4, IL-10, and IL-13) cytokines is essential in the control of inflammation [25]. Here, we monitored a panel of chemokines and cytokines in exudates to assess whether lidocaine specifically regulates their levels. At 4 h after zymosan challenge, most cytokines and chemokines were dramatically up-regulated compared to naïve mice (Fig. 5A). Of interest, both 0.08% and 0.008% lidocaine gave similar results at 4 h, with significant, preferential reduction of anti-inflammatory cytokines, including IL-4, IL-10 and IL-13 (Fig. 5B). In addition, anesthetic dose of lidocaine decreased pro-inflammatory KC (the murine homolog of human IL-8) without significant changes in other pro-inflammatory cytokines and chemokines in the exudates, suggesting that lidocaine acts at several levels in acute inflammation, overall reducing what is coined the “cytokine/chemokine storm” observed in the early inflammatory response (4 h) (Fig. 5A).

In murine peritoneal exudates, lidocaine initially reduced the levels of most of the chemokines and cytokines induced by zymosan at 4 h, but increased their levels by 12 h (Table 2). Calculation of the ratios between pro- and anti-inflammatory cytokines (TNF-α/IL-10 or IL-6/IL-10) indicated that lidocaine increased these ratios in the resolution phase (12 and 24 h after zymosan A injection) (Table 3). These changes in ratios (i.e. pro-/anti-inflammatory cytokines) likely contribute to the increased numbers of PMN present in the resolution phase (i.e. 12-24 h), compared to the mice not treated with lidocaine. We also found that lidocaine (0.08%) significantly decreased exudate levels of TGF-β at the late resolution phase, 48 h after zymosan challenge (Fig. 5C). It is likely that the decreased levels of TGF-β contributed to impaired macrophage phagocytosis in lidocaine-treated mice (Fig. 3B), that can lead to delayed PMN clearance and their increased dwell time. The impact of lidocaine was also evaluated in human whole blood *ex vivo* to access whether the murine system reflects human tissue events. The anesthetic dose of lidocaine (0.08%) significantly diminished the levels of a panel of chemokines and cytokines in zymosan-stimulated human whole blood (Fig. 5D).

**Table 2:** Regulation of cytokines/chemokines by lidocaine in murine exudates

Time	4 h		12 h	
Lidocaine	0.008%	0.08%	0.008%	0.08%
*Pro-inflammatory cytokines*				
IL-1β	+	+	++	– –
IL-6	– – – *	– –	+++	+
IL-12	– –	– –	– – –	– – –
IFN-γ	–	–	++	–
TNF-α	– – – *	– –	+++ *	+
KC	– – – *	– – – *	+++	+
JE	– –	– –	+++ *	++
MCP5	– –	+	++ *	++
MIP-1α	–	– –	+	–
RANTES	– –	+	+	Ø
TARC	–	Ø	+++ *	–
*Anti-inflammatory cytokines*				
IL-4	– – – *	– – – *	+++	+++
IL-10	– –*	Ø	+++	+
IL-13	– – – *	– – – *	+++ *	+
TGF-β	Ø	Ø	Ø	Ø

Mice were administered 1 mg zymosan by intraperitoneal injection in the absence or presence of lidocaine. Peritoneal lavages were obtained at 4 h and leukocytes enumerated. Cell-free fluids were collected and amounts of selected pro-and anti-inflammatory cytokines and chemokines determined by multiplexed sandwich ELISA. Results are expressed as “**Ø**”: no change, “**–**”: 0–20% reduction, “**– –**”: 20–40% reduction, and “**– – –**”: 40–60% reduction; and “**+**”: 0–20% increase, “**++**”: 20–40% increase, and “**+++**”: 40–60% increase of selective cytokine in the presence of lidocaine compared to mice injected with zymosan alone. Results represent mean from n = 3–4. ^*^ *P*≤0.05

### Volatile anesthetic isoflurane promotes resolution

**Figure 6:**
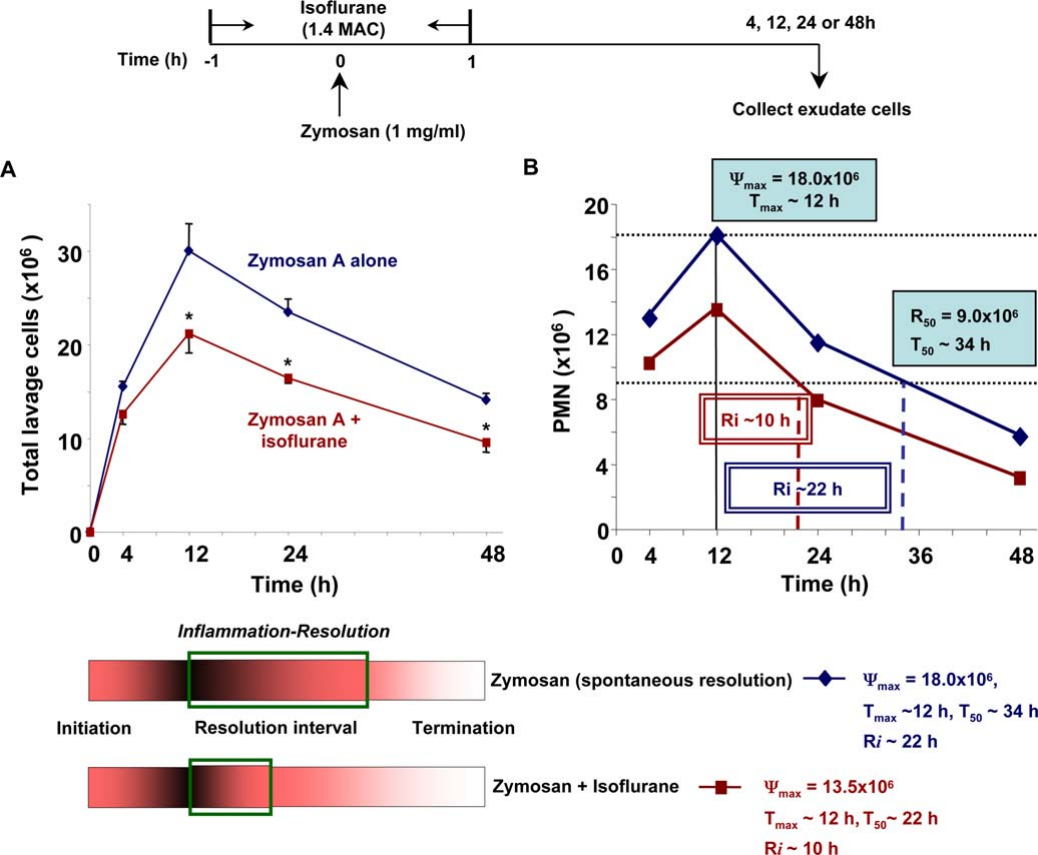
Volatile anesthetic isoflurane reduces leukocyte infiltration and promote resolution by shortening resolution interval. Mice were administered 1.4 MAC of isoflurane one hour prior to and after injection of zymosan A (1mg/ml, i.p.) (see timeline). The peritoneal lavages were collected at indicated time points. (A) Total leukocytes were enumerated by light microscopy, and PMN and mononuclear cells determined by differential leukocyte counting. Results are expressed as the mean±SEM from n = 3-4. *P<0.05 when compared to mice treated with zymosan A alone at the same intervals. (B) Resolution Indices were calculated with isoflurane as in Figure S1. Isoflurane treatment reduces the magnitude (Ψ_max_) of inflammation and accelerates resolution by shortening the resolution interval (R*_i_*.).

Isoflurane 1.4 MAC (minimum alveolar concentration) was administrated over a 2 h period (from 1 h before to 1 h after zymosan challenge) to mimic clinical use. Exudates were collected during inflammation-resolution to determine the potential impact of isoflurane in the resolution maps and indices. Isoflurane significantly reduced zymosan-stimulated leukocyte infiltration at 12, 24 and 48 h (Fig. 6A). When compared to mice that received zymosan alone, at the peak of inflammation, T_max_, isoflurane decreased maximal PMN numbers (Ψ_max_) from 18.0×10^6^ to 13.5×10^6^. In addition, isoflurane dramatically reduced T_50_ from ∼34 h to ∼22 h, thus shortening R*_i_* by >50% from ∼22 h to ∼10 h (Fig. 6B). These results contrast with lidocaine's impact on the resolution indices where lidocaine delayed the onset of resolution, i.e. T_max_ (Table 4).

### Isoflurane specifically regulates key exudate proteins

**Figure 7:**
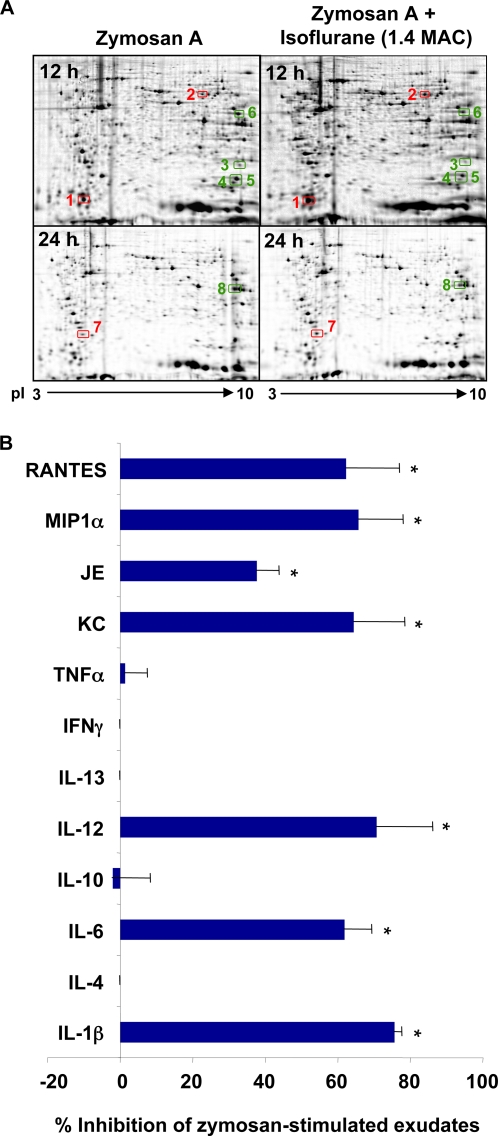
Isoflurane regulates cellular proteins-proteomic analysis. Mice were administered 1.4 Mac of isoflurane one hour prior to and after injection of zymosan A (1mg/ml, i.p.). (A) The peritoneal lavage cells were collected at indicated time points and proteins separated by two-dimensional gel electrophoresis. Changes in individual protein levels were measured by image analysis. Selected proteins that display significant differences between treatments are denoted, and were identified by LC/MS/MS and peptide mapping. (B) Peritoneal cell-free lavage fluids were collected. Cytokine and chemokine levels were determined and expressed as percent inhibition of zymosan A-induced cytokine/chemokine levels by isoflurane. *p<0.05 when compared to mice treated with zymosan A alone. For raw values (pg/ml) of these selective cytokines, see Table 6.

Since isoflurane gave the most dramatic reduction on zymosan–stimulated PMN infiltration at two time intervals (Fig. 6A), these intervals were selected for exudate proteomic analysis: First, the early resolution phase at 12 h, and second, the late resolution phase at 24 h after zymosan challenge (Fig. 7A). At 12 h after zymosan challenge, we found CRAMP, an anti-microbial protein and chemotactic factor was decreased ∼2-fold (Table 5) in mice that received 1.4 MAC isoflurane, contrasting with significant increase of CRAMP at 24 h mediated by lidocaine (Table 1). In addition, isoflurane reduced cofilin-1 (Table 5), a major actin-depolymerization factor regulating actin dynamics and generation and maintenance of cell protrusions, key cellular events that are required for migration [26]. Therefore, during resolution of inflammation, isoflurane selectively regulates cellular proteins that are involved in cell migration and chemotaxis (i.e., CRAMP and cofilin-1). By comparison, isoflurane treatment increased SH3 domain-binding glutamic acid-rich like protein (SH3BGRL), which might have anti-oxidative and anti-inflammatory properties [27], [28].

We monitored in the exudates a panel of chemokines and cytokines to examine whether isoflurane regulates their levels in vivo. Of interest, isoflurane-treated mice selectively reduced zymosan-stimulated pro-inflammatory cytokine levels (IL-1β, IL-6, IL-12, KC, JE [the mouse homolog of human MCP-1], MIP-1α and Rantes) (Fig. 7B, Tables 6 and 7), but did not apparently affect the levels of cytokines IL-4, IL-10 and IL-13 in the early inflammatory phase, 4 h after zymosan challenge (Fig. 7B).

## Discussion

A systems approach to mapping the resolution of acute inflammation demonstrated that resolution is an active process [2], [3] and a new terrain of cellular and molecular processes directed toward returning the tissue to homeostasis [8], [29]. Using this differential-temporal and quantitative systems approach to analyze inflammation and its spontaneous resolution, we identified, for the first time, in the present report that widely used anesthetics impact the resolution of acute inflammation. Lidocaine, the first amino amide-type local anesthetic is well appreciated to affect depolarization in neurons by blocking the fast voltage gated sodium channels on cell membranes [30]. Earlier evidence from in vitro studies indicates that lidocaine influences the immune system by reducing responses such as chemotaxis, microtubule assembly, phagocytosis, release of lysosomal enzymes and superoxide anion generation [31]–[33]. In certain settings, lidocaine can reduce inflammatory responses and protect tissues from local injury [34]. On the other hand, lidocaine worsens renal injury following ischemia-reperfusion by increasing necrosis and local inflammation [35]. In burn wounds, lidocaine increases leukocyte numbers, which suggests an increase in PMN infiltration and/or increased viability of the leukocytes at the burn site [36]. Yet the clinical significance of these observations remains to be established. The results of the present studies demonstrate that lidocaine imposes a molecular lesion in resolution that delays the return to homeostasis. Specifically, lidocaine increases leukocyte accumulation in exudates, impairs the apoptosis of PMN and hampers ingestion of apoptotic PMN by macrophages *in vivo*. Summation of these multi-level actions in tissues significantly delays resolution of inflammation.

In this context, other currently and widely used therapeutic agents also affect resolution of inflammation. Aspirin, for example, by way of initiating biosynthesis of endogenous lipid mediators (i.e., aspirin-triggered epimer of lipoxin A_4_ [ATL] and resolvin E1), promotes resolution [4], [9]. Cyclin-dependent kinase and specific ERK1/2 inhibitors, in comparison, also promote resolution of inflammation by enhancing PMN apoptosis [37], [38]. In contrast, COX or LOX inhibitors, by blocking the biosynthesis of key lipid mediators, dramatically impairs resolution [9], [10]. In the peritoneal cell-free lavages, LXA_4_ appeared in the early inflammatory phase, 4 h after zymosan challenge. PGE_2_, a signal that can activate the full LXA_4_-biosynthetic capacity *in vivo* [17], was present in the peritoneum prior to peritonitis and elevated during the acute inflammatory response. Lidocaine did not alter either the magnitude or time course of LXA_4_ in a statistically significant fashion compared to the mice given zymosan alone. However, a trend towards reduction was observed at 4, 12 and 48 h. Of interest, when exogenous ATLa (a stable analog of LXA_4_ and ATL) was given together with lidocaine, it significantly reversed in part lidocaine's delaying effects in the resolution of inflammation. Thus, pro-resolution mediators may have therapeutic potential in settings where sustained inflammation and impaired resolution are components of disease pathophysiology.

Increases in the ratios of IL-6/IL-10 are thought to signify pro- versus anti-inflammatory response. This increase in ratio correlates with the severity of systemic inflammatory response syndrome and injury after trauma [39]. Since results from several studies indicate that the relationship between pro- and anti-inflammatory cytokines influences the severity of sepsis [40], and TNFα/IL-10 ratios are used as an indicator for disease severity [41], we calculated the ratios of pro- to anti-inflammatory cytokines (Table 3) and found that lidocaine increased these values in the late phase (12 and 24 h after zymosan). Thus, changes in the balance between pro- and anti-inflammatory cytokines, rather than individual chemokines or specific cytokines, appear to contribute to the observed increases in PMN numbers obtained at 24 h with lidocaine.

**Table 3:** Ratios of pro- versus anti-inflammatory cytokines

Time	4 h	12 h	24 h
*TNF-α/IL-10*			
Zymosan A alone	0.5	11.0	43.5
+0.008% lidocaine	0.4	12.5	66.7
+0.08% lidocaine	0.4	11.0	ND
*IL-6/IL-10*			
Zymosan A alone	329.2	236.0	134.0
+0.008% lidocaine	232.7	301.0	177.0
+0.08% lidocaine	254.8	251.0	ND

ND, not determined.

It is noteworthy that, during an inflammatory disease state, a complex network of interactions between different cytokines is likely to occur. The timing of cytokine release and the balance between pro- and anti-inflammatory cytokines is likely to contribute to the overall outcome and severity, as both pro- and anti-inflammatory mediators interact in highly specific ways. Along these lines, computational simulations were carried out to address these complex interactions in the setting of acute inflammation as well as simulate certain disease scenarios and the time course of cytokine levels in mice [42]. This approach may lead to *in silico* development of new therapeutics and real-time diagnostics.

The volatile anesthetic isoflurane binds to gamma-aminobutyric acid type A (GABAA) receptors, glutamate and glycine receptors, and inhibits conduction in activated potassium channels [30]. It is noteworthy that human peripheral mononuclear cells express several GABAA receptor subunits, and application of GABA reduced formyl peptide (fMLP)-stimulated increases in intracellular Ca^2+^ levels [43]. Thus, these GABA receptors may play a role in modulating immune responses. Along these lines, isoflurane is known to impact the inflammatory response, reducing inflammation in vivo [14], increasing leukocyte rolling velocities in mesenteric microcirculation [44], and decreasing activation of the L-selectin and β2-integrins CD11a and CD11b involved in these responses [45]. In the present report, we identified specific proteins in inflammatory exudates and cytoskeleton protein cofilin-1 that were reduced by volatile anesthetics and are known to be important in cell migration. In addition, isoflurane treatment increased SH3BGRL. The human homolog of SH3BGRL belongs to the thioredoxin-like protein superfamily [27]. Among them, thioredoxin-1 (TRX) is a small multifunctional protein with antioxidative and redox-regulating functions [27]. Serum TRX levels were elevated in patients with inflammatory bowel disease. Also, TRX significantly ameliorated DSS-induced colitis and colonic inflammation of IL-10 deficient mice [28]. Thus, SH3BGRL and other thioredoxin-like proteins might have anti-inflammatory properties, and contribute to the accelerated resolution in isoflurane-treated mice documented in the present report (Fig. 6). Of interest, LXA_4_ stimulates IL-10 [9] as well as heme oxygenase-1 [46], [47] and, as indicated in the present report, was able to partially rescue the lidocaine-delayed resolution of inflammation (Fig. 2B).

Isoflurane and lidocaine gave opposite effects in the resolution of acute inflammation, as indicated by their differential impact in the resolution indices (Table 4). This reflects their distinct and selective impact on specific molecules involved in resolution of inflammation. For example, CRAMP protein levels were decreased in mice with isoflurane, contrasting with significant increases in CRAMP at 24 h as evoked by lidocaine, compared with mice given zymosan alone. Thus, it is likely that the reported chemotactic property of CRAMP [22] contributes to the observed opposing actions of isoflurane and lidocaine on peritoneal leukocyte infiltration. Also, isoflurane selectively reduced zymosan-stimulated pro-inflammatory cytokine levels (Fig. 7B and Tables 6 and 7), which contrasts the events in mice treated with zymosan and anesthetic dose of lidocaine (0.08%), that significantly reduced anti-inflammatory IL-13 (Fig. 5B). Hence, the changes in these exudates proteins might reflect the opposing actions of isoflurane and lidocaine in resolution of inflammation.

**Table 4:** Regulation of resolution indices: lidocaine vs. isoflurane

	Ψ_max_ PMN# (x10^6^)	T_max_ (hours)	T_50_ (hours)	R_i_ (hours)
Zymosan-initiated peritonitis	17.5	12	35	23
Zymosan+lidocaine	18.5	24	45	21
Zymosan-initiated peritonitis	18.0	12	34	22
Zymosan+isoflurane	13.5	12	22	10

*Resolution indices are quantitatively defined as:* (i) Magnitude (Ψ_max,_ T_max_)–The time point (T_max_), following challenge or injury, when neutrophil numbers in tissues or exudates reach maximum (Ψ_max_); (ii) Duration (R_50,_ T_50_)–The time point (T_50_) when the neutrophil numbers reduce to 50% of Ψ_max_ (R_50_); (iii) Resolution Interval (R*_i_*)–The time interval from the maximum neutrophil infiltration time point (Ψ_max_) to 50% reduction point (R_50_) [*i.e.* T_50−_T_max_]. Lidocaine treatment enhanced the magnitude (Ψ_max_) and delayed the onset of resolution (T_max_). In contrast, isoflurane reduced magnitude (Ψ_max_) and shortened resolution interval (R*_i_*). See Figure S1 for further details and calculations.

**Table 5:** Cellular proteins regulated by isoflurane during inflammation-resolution

Spot	Protein	Time (h)	# identified peptides	Zymosan A	Zymosan A+Lidocaine	P value
*Cellular protein*						
1	SH3 domain-binding glutamic acid-rich like protein	12	2	10442±752	18668±4356 (↑79%)	0.032
2	Pyruvate kinase M	12	7	1328±200	2104±402 (↑58%)	0.048
3	Transgelin-2	12	5	2228±503	1129±158 (↓50%)	0.023
4	Destrin (ADF) or Cathelicidin (CRAMP)	12	22	4654±190	2327±765 (↓50%)	0.007
5	Cofilin-1	12	2	10040±2177	4367±953 (↓57%)	0.014
6	Aldolase-1	12	13	6138±1065	3730±624 (↓40%)	0.028
7	Neutrophilic granule protein	24	3	2139±410	4483±318 (↑110%)	0.001
8	Similar to GAPDH	24	2	11059±1593	4336±1632 (↓61%)	0.007

The proteins were identified by mass spectrometry (see Materials and Methods).

**Table 6:** Regulation of cytokines: lidocaine vs. isoflurane (4h after zymosan challenge)

Treatment	Zymosan A	+Lidocaine (0.08%)	Zymosan A	+Isoflurane (1.4 MAC)
*Pro-inflammatory cytokines (pg/ml)*				
IL-1β	65.0±7.4	72.3±14.8	170.0±30.4	41.4±3.5*
IL-6	38183.7±6291.8	27391.4±6397.5	65093.3±12314.0	24808.3±4953.4*
IL-12	11.2±2.7	8.6±2.0	9.1±1.6	2.7±1.4*
TNF-α	60.5±3.0	44.9±6.1	128.6±10.6	126.8±8.0
KC	4489.0±553.7	1808.5±306.5*	6311.7±961.5	2243.1±890.0*
JE	5074.3±523.3	3765.3±319.6	8518.3±802.0	5308.3±535.4*
MIP-1α	137.0±13.5	98.5±17.6	173.5±21.9	59.4±21.5*
RANTES	42.8±4.7	45.9±5.9	31.7±5.8	11.9±4.7*

* Significant inhibition of zymosan-stimulated cytokine/chemokine levels

**Table 7:** Regulation of cytokines: lidocaine vs. isoflurane (% inhibition)

Treatment	+Lidocaine (0.08%)	+Isoflurane (1.4 MAC)
*Pro-inflammatory cytokines (% inhibition by anesthetics)*		
IL-1β	–	↓ 75.6% *
IL-6	–	↓ 61.9% *
IL-12	–	↓ 70.8% *
TNF-α	–	–
KC	↓ 60.7% *	↓ 64.5% *
JE	–	↓ 27.7% *
MIP-1α	–	↓ 65.8% *
RANTES	–	↓ 62.3% *

↓^*^ Significant inhibition of zymosan-stimulated cytokine/chemokine levels

**–** No significant change

Historically, the phagocytic index was defined in the cellular era and was used to determine the average number of bacteria ingested by phagocyte at single-cell level [48], [49]. By comparison, the resolution indices presented here expand the appreciation of the complexity of phagocytes at the tissue level and account for the summation of multi-level cellular and molecular events during resolution of inflammation. In conclusion, the results of the recent study indicate that the local anesthetic lidocaine delays the onset of resolution. The impact of lidocaine is documented herein at multi-levels in resolution and reflects (i) increased exudate PMNs, (ii) impaired PMN apoptosis as well as their uptake by macrophages, (iii) modulating both pro- and anti-inflammatory proteins, including cytokines and chemokines. Dysregulation of resolution programs by lidocaine may have important unwanted consequences in both immune responses and host defense that were previously unappreciated.

Clinical implications of the present observations might be far-reaching. Every serious surgical intervention unavoidably results in an inflammatory response. Severity of many postoperative surgical complications, particularly infection, are directly related to the degree and length of inflammatory response and resolution of such response during the postoperative period; therefore, hypothetically, accelerating resolution of postoperative inflammation should be helpful in the management of surgical patients during the postoperative period. Resolution of inflammation would become a resolution of many problems surgical patients face daily. The results of the present study offer new avenues, not only for continued studies in the cellular and molecular markers in resolution of inflammation, but also for future translational and clinical research. Also, combining pro-resolution molecules, such as LXA_4_ and ATL, together with lidocaine may be a useful strategy to rescue resolution of acute inflammation. In sharp contrast, the volatile anesthetic isoflurane accelerates resolution, shortening resolution interval. Together, these findings demonstrate, for the first time, the direct impact of anesthetics in the resolution of inflammatory challenge and the return of the local tissue to homeostasis.

## Materials and Methods

### Murine acute inflammation

For lidocaine treatment, male FVB mice (6-8 weeks; Charles River, Wilmington, MA) were administered lidocaine (0.08% or 0.008%) intraperitoneally together with 1 mg/ml zymosan A (i.p.) to evoke peritonitis [8] as in accordance with the Harvard Medical Area Standing Committee on Animals (protocol no. 02570). ATLa (a stable analog of aspirin-triggered LXA_4_) was prepared by total organic synthesis in the Organic Synthesis Core (P50-DE016191). For isoflurane treatment, mice were administered 1.4 MAC [50] of isoflurane for a 2 h period (from 1h before to 1 h after injection of zymosan, i.p.) (see timeline in Fig. 6). At indicated time points, mice were euthanized with an overdose of isoflurane, and peritoneal exudates were collected by lavaging with 5 ml sterile saline. Exudate cells and supernatants were obtained for analyses described below.

### Human whole blood

Venous blood (anticoagulated with 10 U/ml sodium heparin) was collected from healthy non-smoking volunteers who declared not to have taken any drugs for at least two weeks before the experiments. Informed consent was obtained from each volunteer. The protocol was approved by the Brigham and Women's Hospital Institutional Review Board (protocol no. 88-02642, approved 11/26/07). Heparinized whole blood was then incubated with either 0.008% or 0.08% of lidocaine in the presence of zymosan A (100 µg/ml) for 4 h, and plasma was collected by centrifugation at 2,000 rpm for 15 min. The amounts of cytokines and chemokines levels were determined by multiplexed sandwich ELISA (SearchLight Proteome Array custom-designed by Pierce Boston Technology Center). Following a standard sandwich ELISA procedure, the entire plate is imaged to capture chemiluminescent signals generated at each spot within each well of the array. The SearchLight CCD Imaging and Analysis System features image analysis software that calculates chemokine/cytokine concentrations (pg/ml) using pre-determined standard curves.

### Differential leukocyte counts and FACS analysis

Aliquots of exudate cells were prepared for determination of total and differential leukocyte counts. For determination of cellular composition (PMN vs. mononuclear cells), cells were blocked with anti-mouse CD16/32 blocking Ab (0.5 µg/0.5×10^6^ cells) for 5 min and stained (20 min) with FITC-conjugated anti-mouse CD14 and PE-conjugated anti-mouse Ly-6G (0.5 µg/0.5×10^6^ cells; clones rmC5-3 and RB6-8C5, respectively, from BD Pharmingen, San Diego, CA). FACS analysis was then carried out.

### Apoptosis and phagocytosis

For determining PMN apoptosis *in vivo*, exudate cells were labeled with FITC-conjugated anti-annexin-V Ab (0.5 µg Ab/0.5×10^6^ cells, eBioscience) and PE-conjugated anti-mouse Gr-1 (Ly-6G) Ab (0.5 µg Ab/0.5×10^6^ cells, eBioscience) for 20 min. The annexin-V^+^Gr-1^+^ PMN population was determined by FACS.

For determining macrophage phagocytosis of apoptotic PMN *in vivo*, cells were blocked with anti-mouse CD16/32 blocking Ab (0.5 µg/0.5×10^6^ cells) for 5 min, stained with FITC-conjugated anti-mouse F4/80 (0.5 µg/0.5×10^6^ cells) for 20 min, and then permeabilized with 0.1 % Triton X-100 (100 µl, 10 min). Permeabilized cells were then stained with PE-conjugated anti-mouse Ly-6G (0.5 µg/0.5×10^6^ cells). The F4/80^+^Gr-1^+^ cell population was determined by FACS.

For phagocytosis *in vitro*, murine peritoneal resident macrophages were collected and plated onto 24-well plates (1×10^5^cells/well) and incubated with lidocaine (0.008% or 0.08%), LXA_4_ (1 nM; Calbiochem) or both for 20 min. FITC-zymosan (2.5 µl/well) was then added to macrophages for 30 min. Supernatant was aspirated and extracellular fluorescence was quenched by adding trypan blue for 1 min. Cells were then washed and intracellular fluorescence was determined by a fluorescent plate reader.

### Two-dimensional gel-based proteomics

#### Two-dimensional gel electrophoresis

Supernatants and cell pellets from peritoneal lavages were collected by centrifugation (15 min, 1,800 rpm) in the presence of protease inhibitors (Roche, Indianapolis, IN). Proteins in the supernatant were desalted by acetone precipitation, and the protein pellet was re-dissolved in a lysis solution containing 8 M urea, 4% w/v CHAPS, 40 mM Tris, and 65 mM dithiothreitol (DTT). The cell pellets were directly solubilized in the same lysis solution through sonication at 4°C. The protein concentrations were measured in duplicate by a Bradford protein assay kit (Bio-Rad, Hercules, CA) in a 96-well plate format using bovine serum albumin as the standard. Supernatant (25 µg) or cellular (50 µg) proteins from each animal were mixed with 125 µL of rehydration buffer containing 8M urea, 2% (w/v) CHAPS, 10 mM DTT, and 0.2% carrier ampholytes (pH 3–10), and then loaded onto nonlinear 7-cm, pH 3-10, IPG strips (Bio-Rad) through passive in-gel rehydration overnight. After iso-electric focusing for 10,000 V-h, the proteins in the IGP strips were reduced with dithiothreitol and alkylated with iodoacetamide. The 2^nd^ dimension separation was then carried out using 10–14% SDS-PAGE (covering ∼MW10 to 200 kDa). Gels were stained with ProteomIQ™ blue dye (Proteome Systems, Woburn, MA), and scanned with a GS-800 densitometer system (Bio-Rad). Image analysis was carried out with PDQuest software (version 8.0) (Bio-Rad). The differentially regulated protein spots were selected based on the normalized spot volumes.

#### LC-MS-MS proteomics

The selected protein spots were excised and in-gel digested with sequencing grade trypsin (Promega, Madison, WI). Tryptic peptides were loaded onto a 2 µg capacity peptide trap (CapTrap; Michrom Bioresources, Auburn, CA) in 0.1% formic acid and 0.05% trifluoroacetic acid and separated by capillary liquid chromatography using a capillary column (75 µm×5 cm×3 µm; LC Packings, Amsterdam, The Netherlands) at 150 nl/min delivered by an Agilent 1100LC pump (400 µl/min) and a flow splitter (Accurate, LC Packings). A mobile phase gradient was run using mobile phase A (2% acetonitrile/0.1% formic acid), and B (80% acetonitrile/0.1% formic acid) from 0–10 min with 0–20% B followed by 10–90 min with 20–60% B. Peptide mass and charge was determined on a ThermoFinnigan Advantage ion-trap mass spectrometer (San Jose, CA) after electrospray ionization using end-coated spray Silicatip tip (ID 75 µm, tip ID 15 µm, New Objective) held at a spray voltage of 1.8 kV. After acquisition of the peptide parent ion mass, zoom scans and tandem mass spectra of parent peptide ions above a signal threshold of 2×10^4^ were recorded with dynamic exclusion, using Xcalibur 1.3 data acquisition software (ThermoFinnigan).

#### Protein identification

Proteins were identified by peptide mapping of tryptic peptide tandem mass spectra using TurboSequest (BioWorks 3.1 software, ThermoFinnigan against indexed Swiss-Prot protein database). Protein modifications that were taken into consideration included methionine oxidation and alkylation of cysteine with iodoacetamide. The search results were filtered by X_corr_ vs. charge with 1.5 for singly charged ions, 2.0 for doubly charged ions, and 2.5 for triply charged ions. A protein was considered identified when a minimum of two tryptic peptides were matched.

### ELISA-peptide and lipid mediators

Aliquots of supernatants were used to quantitate chemokines and cytokines using a SearchLight Mouse Chemokine Array custom designed with Pierce Boston Technology Center. TGF-β levels was determined with ELISA using a monoclonal anti-TGF-β antibody (R&D Systems, Minneapolis, MN) recognizing the active forms of TGF-β (1, 2, and 3). Eicosanoid ELISAs (LTB_4_, LXA_4_ and PGE_2_) were carried out following manufacturer's instructions (Neogen, Lexington, KY).

### Western blot

Supernatants from peritoneal lavages were collected and equal amounts of proteins were subjected to SDS-PAGE and transferred to a polyvinylidene fluoride (PVDF) microporous membrane by electroblotting. Membranes were blocked in 5% non-fat milk in TBST (0.9% NaCl and 0.05% Tween-20 in 20 mM Tris/HCl, pH 7.4) and probed with a goat anti-mouse S100A9 polyclonal antibody (0.2 µg/ml, R&D Systems) for 1 hour. After washing three times with TBST, membrane were incubated with HRP-linked anti-goat IgG (1:5,000 dilution) for 1 h and the immunoreactive bands were developed by incubating with chemiluminescence substrates and visualized by exposure to an X-ray film.

### RT-PCR

Murine peritoneal cells were collected, total RNA was isolated using TriZol reagent (GIBCO BRL, Grand Island, NY) and reverse-transcribed followed by polymerase chain reactions (PCR) using HotStar Master mix (Qiagen) (95°C for 15 min, then 35 cycles of 95°C for 30 sec, 55°C for 30 sec and 72°C for 60 sec) with specific primers for mouse S100A9 (sense: 5′-CCCTGACACCCTGAGCA AGAAG-3′ and antisense 5′-TTTCCCAGAACAAAGGCCATTGAG-3′). Relative intensities of RT-PCR products were quantified and normalized by β-actin message levels using the public domain NIH image program (developed at the NIH, available on the Internet).

### Statistical approaches

All results were calculated and expressed as mean±standard error of mean (mean±SEM). Group comparisons were carried out using one-way ANOVA or Student's *t*-test where appropriate, with P values <0.05 taken as statistically significant (sufficient to reject the null hypothesis).

## Supporting Information

Figure S1 (A) *Resolution indices: definitions and calculations*. The main events in the resolution of acute inflammation can be quantified [8] with the introduction of resolution indices defined as (i) Magnitude (Ψ_max_, T_max_)–The time point (T_max_), following challenge or injury, when neutrophil numbers in tissues or exudates reach maximum (Ψ_max_); (ii) Duration (R_50_, T_50_)–The time point (T_50_) when the neutrophil numbers reduce to 50% of Ψ_max_ (R_50_); (iii) Resolution Interval (R*_i_*)–The time interval from the maximum neutrophil infiltration time point (Ψ_max_) to 50% reduction point (R_50_) [*i.e*. T_50_-T_max_]. For calculating specific resolution indices and further details, see ref. 8. (B) *Lidocaine treatment alone*. Mice were injected with lidocaine (0.08%) or saline and peritoneal lavages were collected 4 and 24 h after injection. Total leukocytes were enumerated by light microscopy. Results are expressed as the mean of two separate experiments. (C) *Resolution Indices calculated with lidocaine*. Lidocaine treatment enhances the magnitude (Ψ_max_) and delays the onset (T_max_) of resolution.
(2.38 MB TIF)
