# Supplementary figures and images for: Anesthetics Impact the Resolution of Inflammation
Source: PLoS One. 2008 Apr 2;3(4):e1879. doi: 10.1371/journal.pone.0001879 (PMC2268966; doi:10.1371/journal.pone.0001879)

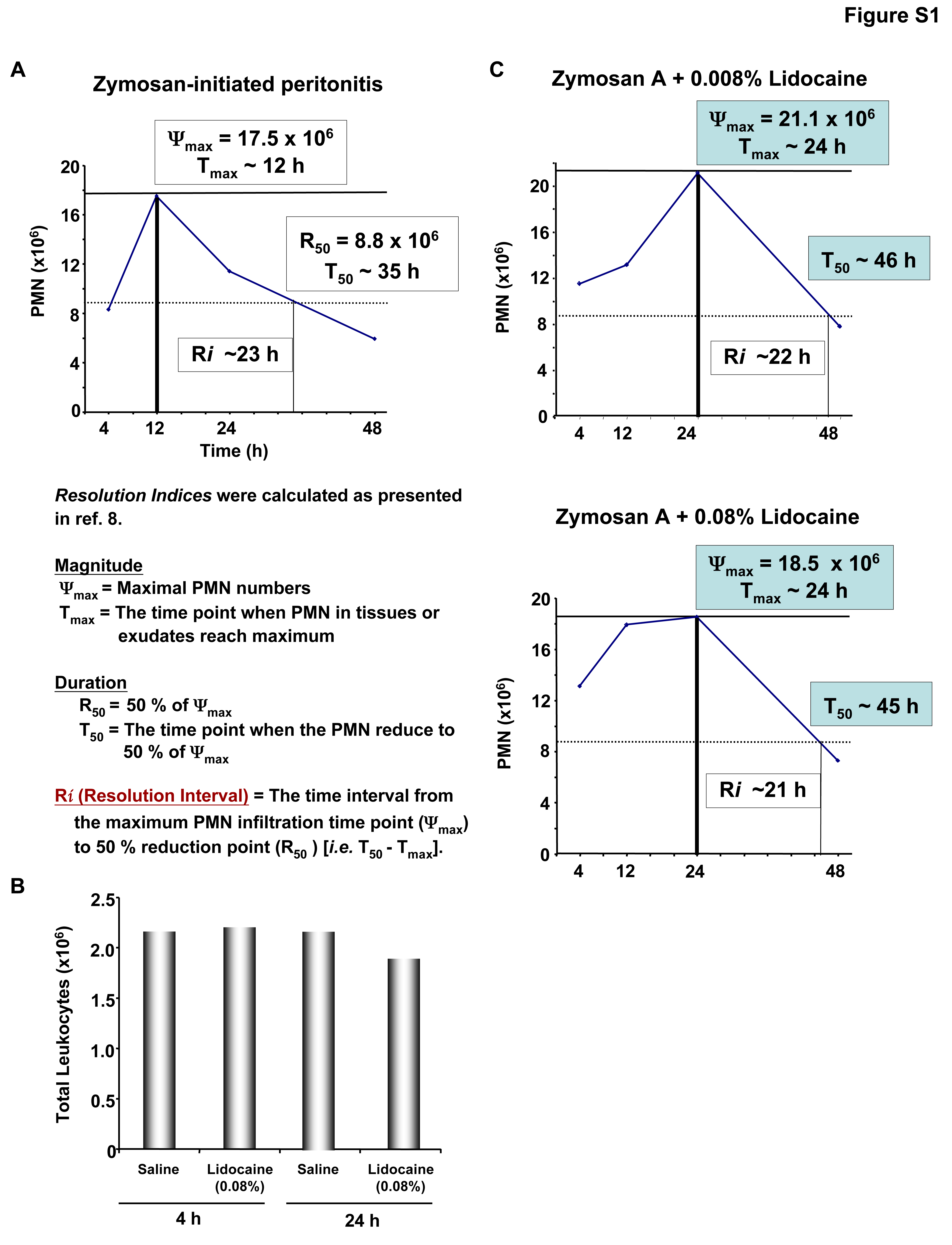

Supplement: Figure S1 — (A) Resolution indices: definitions and calculations. The main events in the resolution of acute inflammation can be quantified [8] with the introduction of resolution indices defined as (i) Magnitude (Ψmax, Tmax)–The time point (Tmax), following challenge or injury, when neutrophil numbers in tissues or exudates reach maximum (Ψmax); (ii) Duration (R50, T50)–The time point (T50) when the neutrophil numbers reduce to 50% of Ψmax (R50); (iii) Resolution Interval (Ri)–The time interval from the maximum neutrophil infiltration time point (Ψmax) to 50% reduction point (R50) [i.e. T50-Tmax]. For calculating specific resolution indices and further details, see ref. 8. (B) Lidocaine treatment alone. Mice were injected with lidocaine (0.08%) or saline and peritoneal lavages were collected 4 and 24 h after injection. Total leukocytes were enumerated by light microscopy. Results are expressed as the mean of two separate experiments. (C) Resolution Indices calculated with lidocaine. Lidocaine treatment enhances the magnitude (Ψmax) and delays the onset (Tmax) of resolution. (2.38 MB TIF) [file pone.0001879.s001.tif]
